# Managing relapsed refractory lymphoma with palliative oral chemotherapy: A multicentre retrospective study

**DOI:** 10.1002/jha2.537

**Published:** 2022-09-02

**Authors:** Simon J. Bulley, Anna Santarsieri, Isabel C. Lentell, Brendan O'Sullivan, Andrew Hodson, Oliver Firth, Shalal Sadullah, Annabel M. Follows, Mamatha Karanth, Sandra Young Min, Alexis Fowler, James Russell, Benjamin J. Uttenthal, Daniel J. Hodson, George A. Follows

**Affiliations:** ^1^ Department of Haematology and Pharmacy Cambridge University Hospitals NHS Foundation Trust, Cambridge Biomedical Campus Cambridge UK; ^2^ Murray Edwards College, University of Cambridge Huntingdon Road Cambridge UK; ^3^ Pharmacy Department Cambridge University Hospitals NHS Foundation Trust, Cambridge Biomedical Campus Cambridge UK; ^4^ Department of Haematology Ipswich Hospital Ipswich Suffolk UK; ^5^ Department of Haematology James Paget University Hospitals NHS Foundation Trust Great Yarmouth Norfolk UK; ^6^ Department of Haematology West Suffolk NHS Foundation Trust Bury St Edmunds Suffolk UK; ^7^ Department of Haematology Hinchingbrooke Hospital North West Anglia NHS Foundation Trust Hinchingbrooke Huntingdon UK; ^8^ Wellcome‐MRC Cambridge Stem Cell Institute University of Cambridge Puddicombe Way Cambridge UK; ^9^ Department of Haematology University of Cambridge Cambridge UK

## Abstract

PEP‐C (prednisolone, etoposide, procarbazine and cyclophosphamide) is an orally administered daily chemotherapy regimen used with palliative intent in relapsed refractory lymphoma. To our knowledge, no data on PEP‐C have been reported since the original group described the regimen. Here we present a multicentre retrospective cohort reporting our use of PEP‐C in 92 patients over an 8‐year period. We find that even heavily pretreated lymphoma can respond to PEP‐C, particularly low‐grade lymphoma (including mantle cell) and lymphoma that was sensitive to the previous line of systemic therapy (chemosensitive). These characteristics may help in the selection of patients likely to derive benefit. The median overall survival of patients with chemosensitive lymphoma treated with PEP‐C is 217 days. Within the limitations of a retrospective cohort, we find that PEP‐C is well tolerated: the most common toxicity leading to discontinuation is marrow suppression. We suggest that PEP‐C should be considered for patients with relapsed refractory lymphoma in two settings: first, where there is no licensed alternative; and second, where the licensed alternative is an intravenous drug and the patient would prefer to choose an oral chemotherapy option.

1

To the Editor:

The choice of therapy in relapsed or refractory (R/R) lymphoma in adults is dependent upon lymphoma subtype, previous treatment, disease response and individual patient factors such as performance status (PS). Some patients will benefit from salvage chemotherapy and selected patients proceed to autologous stem cell transplantation (ASCT) depending on lymphoma subtype and response to therapy. Chimeric antigen receptor (CAR) T‐cell therapy is emerging as a treatment option for highly selected patients with R/R non‐Hodgkin lymphoma (NHL). In addition there are recently approved regimens for specific subtypes of relapsed lymphoma. These include polatuzumab vedotin with rituximab and bendamustine [1], and tafasitamab with lenalidomide [2], both licensed for the treatment of R/R diffuse large B‐cell lymphoma. Pixantrone is licensed for the treatment of multiply R/R high‐grade B‐cell NHL (HGBL) [3] as is ibrutinib for the treatment of R/R mantle cell lymphoma (MCL) [4]. Brentuximab [5], nivolumab [6] and pembrolizumab [7] are licensed in R/R classical Hodgkin lymphoma. There are many patients, however, who have either responded inadequately to these therapies or whose PS or co‐morbidities preclude their use, particularly based on PS restrictions within NHS Blueteq criteria. These patients have a poor prognosis but may benefit from palliative chemotherapy to prolong life and/or provide relief from disease‐related symptoms. Given the palliative intent of such therapy, quality of life and tolerability of treatment are crucial considerations. However, there remain few published studies reporting the efficacy or tolerability of palliative oral chemotherapy options and the combination regimens used vary from centre to centre. These include DECC (dexamethasone, etoposide, chlorambucil and lomustine) [8], CEP (lomustine, etoposide, prednisolone) [9] and COCKLE (lomustine, cyclophosphamide, etoposide, prednisolone) [10] for patients with relapsed/refractory Hodgkin lymphoma or NHL.

PEP‐C (prednisolone, etoposide, procarbazine, cyclophosphamide) is a palliative chemotherapy regimen used in the relapsed/refractory setting for both MCL [11] and other lymphoma subtypes [12]. It benefits from being an oral regimen administered on an outpatient basis. No data have been published regarding PEP‐C since the initial reports of the Weill Cornell/New York Presbyterian group in 2008 [11, 12]. In the East of England (EoE) Lymphoma Network we have been using PEP‐C for relapsed/refractory lymphoma since 2009 and here we present the largest real‐world data set of patients on palliative oral chemotherapy. The objective of this study is to provide evidence on the efficacy and tolerability of PEP‐C, which can then be used to inform patients who are considering palliative chemotherapy options.

Ninety‐two patients commenced treatment with PEP‐C between February 2009 and March 2017 at five EoE centres. Eligibility was determined by real‐world clinical decision making, where PEP‐C was considered effective palliative care. A total of 96 patients were considered eligible, but two died before starting treatment and the records were unavailable for two patients. Patient characteristics on commencing PEP‐C therapy are shown in Table S1. Of 92 patients, 61 (67%) had ‘high‐grade lymphoma’ (32% DLBCL, 26% other HGBL, 5% T‐cell lymphoma, 3% Hodgkin lymphoma) and 31 were classed as ‘low‐grade lymphoma’ (25% MCL, 8% low‐grade B‐cell NHL and 1 myeloid malignancy).

The median age at the start of PEP‐C treatment was 73 years. Patients had received a median of two prior lines of chemotherapy and 11% had received ASCT (Supplementary Table S1). Of the DLBCL and other HGBCL patients, 83% had received R‐CHOP/R‐CVP‐based chemotherapy and 40% had proceeded to intensive salvage chemotherapy. Of the mantle cell lymphoma patients, 4 (17%) had received treatment with ibrutinib. Thirty six per cent of patients were refractory to their most recent line of chemotherapy prior to PEP‐C (chemoresistant disease). At the time of commencing PEP‐C, 91% of patients had stage 3 or 4 disease, 38% had more than one extranodal site of disease and 24% had a PS of 3 or 4. PS data were missing for 27% of the cohort.

Typically patients had 21 days of PEP‐C induction (prednisolone 20 mg after breakfast, cyclophosphamide 50 mg after lunch, etoposide 50 mg after evening meal and procarbazine 50 mg at bedtime). Maintenance treatment was generally given on 3 days a week; however, there was considerable variation in dose requirement. In three of five centres treatment was administered as described by Coleman et al. [11, 12]. In two centres, alternative regimens were used, accounting for 19 of 92 patients. There was no significant effect of treatment centre on the reported outcomes so the data have been pooled. Median duration of treatment with PEP‐C was 76 days (IQR 28 to 159), and the drug cost of the entire course of treatment for the ‘median’ patient would be £557.67 [13].

The median overall survival from starting PEP‐C was 163 days (95% CI: 95 to 230, Figure 1A) with a median time to end‐of‐study of 3.2 years. Within this period of follow‐up 82 patients finished treatment with PEP‐C and 70 patients died.

**FIGURE 1:**
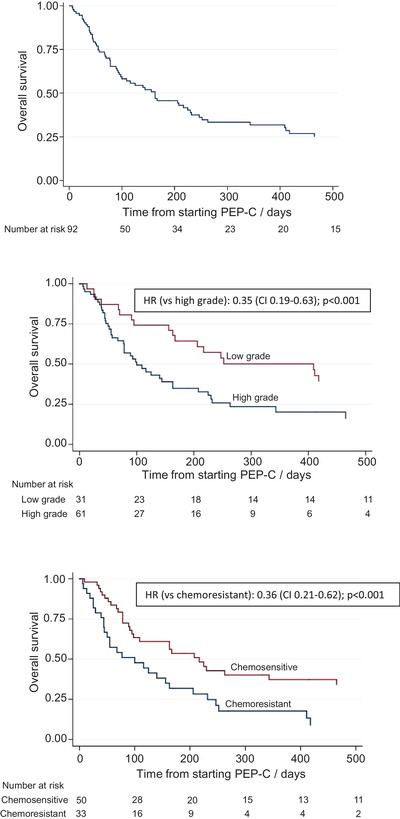
Kaplan‐Meier survival estimate of overall survival from starting treatment with PEP‐C. (A) All patients. Median overall survival = 163 days (95% CI: 95 to 230). (B) Survival estimates grouped by histological diagnosis. Patients with low‐grade disease had a median overall survival of 409 days (95% CI: 163 to 599) versus 100 days (95% CI: 77 to 163) for patients with high‐grade disease. HR = 0.35 (95% CI: 0.19 to 0.63); *p* < 0.001. (C) Survival estimates grouped by the response a patient made to the line of systemic treatment prior to PEP‐C. Patients who were chemosensitive had a median overall survival of 217 days (95% CI: 98 to 465) versus 100 days (95% CI: 45 to 163) for patients who were chemoresistant. HR = 0.36 (95% CI: 0.21 to 0.62); *p* < 0.001

Response to treatment was assessed based on clinical and radiological data, following the revised response criteria for malignant lymphoma [14] (Supplementary Table S2). Sixty one per cent of patients with low‐grade lymphoma achieved a complete or partial response to PEP‐C, compared with 33% with high‐grade lymphoma (odds ratio [OR] = 0.17 [95% CI: 0.05 to 0.60]; *p* = 0.006). Similarly, 50% with chemosensitive disease responded to PEP‐C while only 27% with chemoresistant disease responded to PEP‐C (OR = 0.27 [95% CI: 0.08 to 0.91]; *p* = 0.035). Grade of lymphoma and chemosensitivity also held up as predictors of overall survival on multivariate analysis, as did stage of disease. Median overall survival with low‐grade lymphoma was 409 days versus 100 days with high‐grade lymphoma (hazard ratio [HR] = 0.35 [95% CI: 0.19 to 0.63]; *p* < 0.001) (Figure 1B). For chemosensitive patients, the median overall survival was 217 days versus 100 days for chemoresistant patients (HR = 0.36 [95% CI: 0.21 to 0.62]; *p* < 0.001) (Figure 1C). For patients with stage 4 disease, the median overall survival was 100 days (95% CI: 78 to 163) compared to 252 days (95% CI: 163 to 535) in patients with non‐stage 4 disease (HR 2.22 [95% CI: 1.24 to 3.82]; *p* = 0.007).

Twenty‐four patients (29%) discontinued PEP‐C due to toxicity. Ten patients stopped treatment due to uncomplicated bone marrow suppression, six stopped due to neutropenic sepsis and five due to non‐neutropenic sepsis. The remaining three toxicities were one case each of fungal pneumonia, nausea and a transient ischaemic attack. The notes of 59 patients were available for more detailed assessment of toxicities and the results are summarised in Table 1.

**TABLE 1:** Patient characteristics when starting treatment with PEP‐C and details of PEP‐C treatment

Patient characteristics when starting PEP‐C and details of PEP‐C treatment	*N* = 92
Median age, years (IQR) (range)	73 (67–80) (30–90)
Lymphoma subtypes	
DLBCL	29 (32%)
Other high‐grade B‐cell NHL	24 (26%)
Mantle cell lymphoma	23 (25%)
Low‐grade B‐cell NHL	7 (8%)
T‐cell lymphoma	5 (5%)
Classical Hodgkin's disease	3 (3%)
Myeloid malignancy	1 (1%)
ECOG performance status	
0–2	45 (49%)
3–4	22 (24%)
Missing	25 (27%)
Ann Arbor stage	
1–2	8 (9%)
3–4	84 (91%)
Number of extranodal sites	
0–1	57 (62%)
>1	35 (38%)
Median duration of disease from initial diagnosis, months (IQR)	28 (14 to 64)
Median length of PEP‐C induction treatment, days (IQR)	21 (14 to 29)
Median length of break from PEP‐C before starting maintenance, days (IQR)	0 (0 to 8)
Median maintenance dose, days per week of PEP‐C (IQR)	3 (3 to 3.5)
Median duration of treatment, days with PEP‐C (IQR)	76 (28 to 159)
Reason PEP‐C discontinued	Toxicity (24), refractory disease (21), death (18), completed treatment (10), relapse (6)
Toxicities	*N* = 59 patients, 113 toxicities
Uncomplicated marrow suppression	21 (18%)
Neutropenic sepsis	10 (9%)
Viral reactivation	3 (3%)
Other infections	31 (27%)
Gastrointestinal	21 (18%)
Fatigue	7 (6%)
Respiratory	4 (4%)
Cardiac	2 (2%)
Hair loss	2 (2%)
Bleeding	2 (2%)
Other	10 (9%)

DLBCL, diffuse large B‐cell lymphoma; NHL, non‐Hodgkin's lymphoma; FL, follicular lymphoma; CLL, chronic lymphocytic leukaemia; SLL, small lymphocytic lymphoma; ECOG, Eastern Cooperative Oncology Group.

In conclusion, PEP‐C is a cost‐effective, exclusively oral chemotherapy regimen for relapsed/refractory lymphoma that can be delivered on an outpatient basis. Our results show that it has activity in both low‐ and high‐grade lymphoma. Outcomes and toxicity appear similar to those reported for other palliative regimens.

## AUTHOR CONTRIBUTIONS

S.J.B., I.C.L. and G.A.F. designed the study. S.J.B., I.C.L., B.O'S., A.H., O.F., S.S., A.M.F., M.K., S.Y.M., A.F., J.R., B.J.U. and G.A.F. performed data collection. S.J.B. performed data analysis. S.J.B., A.S., D.J.H., I.C.L. and G.A.F. wrote the manuscript. S.J.B., I.C.L., B.O'S., A.H., O.F., S.S., A.M.F., M.K., S.Y.M., A.F., J.R., A.S., B.J.U., D.J.H. and G.A.F. reviewed the manuscript.

## FUNDING INFORMATION

The article is published open access as per the agreement between University of Cambridge and Wiley.

## ETHICS STATEMENT

This study was registered and approved by the Cambridge University Hospitals Audit department as a retrospective service evaluation on 14 February 2017.

## Supporting information

TABLE S1 Patient characteristics at the time of the initial diagnosis of lymphoma and details of treatment received prior to PEP‐C.

TABLE S2 Response to PEP‐C for all patients and subdivided by patient characteristics found to be statistically significant in a multivariate logistic regression model. A patient is considered to be ‘refractory’ to PEP‐C or to his previous line of chemotherapy (‘chemoresistant’) if a CR or PR has not been achieved and the response has been satisfactorily assessed. ‘Previous line of chemotherapy’ refers to the most recent course of systemic treatment given to the patient prior to PEP‐C. The presence or absence of extranodal disease refers to disease status when starting treatment with PEP‐C. CR, complete response; PR, partial response.

## Data Availability

Due to confidentiality agreements, the source of the data cannot be made available.
