# Supplementary material for: Managing relapsed refractory lymphoma with palliative oral chemotherapy: A multicentre retrospective study
Source: EJHaem. 2022 Sep 2;3(4):1316–20. doi: 10.1002/jha2.537 (PMC9713053; doi:10.1002/jha2.537)
Supplement: Supplementary file 1 — TABLE S1 Patient characteristics at the time of the initial diagnosis of lymphoma and details of treatment received prior to PEP‐C. [file JHA2-3-1316-s001.docx]

**Supplementary table I**

**Patient characteristics at the time of the initial diagnosis of lymphoma and details of treatment received prior to PEP-C.**

Note that all transplants in the table are autologous transplants. If a patient did not make a complete or partial response to their most recent line of chemotherapy prior to PEP-C the response is recorded as ‘refractory’. IQR=Interquartile range. PBSC/BM=Peripheral blood stem cell / bone marrow.

**Supplementary table I**

| Patient characteristics at time of initial lymphoma diagnosis and details of treatment prior to PEP-C | N=92 |
| --- | --- |
| Median age, years (IQR) | 69 (63 to 78) |
| Sex  Female  Male | 37 (40%)  55 (60%) |
| International prognostic index  0-2  ≥3  Missing | 19 (21%)  44 (48%)  29 (32%) |
| Lymphoma subtypes  See table I in main text |  |
| Median number of lines of systemic treatment prior to PEP-C (IQR) (range)  Median number of responses to systemic treatment prior to PEP-C | 2 (1 to 3.5) (0 to 8)  1 (1 to 2) (0 to 6) |
| Previously received PBSC/BM transplant | 10 (11%) |
| Response to most recent systemic chemotherapy prior to PEP-C  Complete or partial  Refractory  Missing data  PEP-C first line treatment | 50 (54%)  33 (36%)  6 (7%)  3 (3%) |
| Number of patients with a response to a line of systemic treatment prior to PEP-C lasting >24 weeks | 66 (72%) |
| Prior therapies in patients with DLBCL and other high-grade B-cell NHL (n=53)  R-CHOP/R-mini-CHOP  R-CVP/R-Gem-CVP  R-DA-EPOCH  Chlorambucil + Prednisolone  ABVD  First-line PEP-C  PMitCEBO  Intensive salvage chemotherapy with R-DHAP/R-Gem-Cis/  R-IVE/R-ICE/ESHAP  Prior therapies in patients with mantle cell lymphoma (n=23)  Rituximab + Bendamustine  Fludarabine + Cyclophosphamide  R-CHOP  High dose cytarabine  Ibrutinib  Chlorambucil  R-DHAP  R-Gem-Cis  Bortezomib  Prednisolone  First-line PEP-C | 37 (70%)  7 (13%)  4 (8%)  2 (4%)  1 (2%)  1 (2%)  1 (2%)  21 (40%)  5 (22%)  8 (35%)  9 (39%)  5 (22%)  4 (17%)  4 (17%)  2 (9%)  1 (4%)  1 (4%)  1 (4%)  3 (13%) |
