# Supplementary material for: Managing relapsed refractory lymphoma with palliative oral chemotherapy: A multicentre retrospective study
Source: EJHaem. 2022 Sep 2;3(4):1316–20. doi: 10.1002/jha2.537 (PMC9713053; doi:10.1002/jha2.537)
Supplement: Supplementary file 2 — TABLE S2 Response to PEP‐C for all patients and subdivided by patient characteristics found to be statistically significant in a multivariate logistic regression model. A patient is considered to be ‘refractory’ to PEP‐C or to his previous line of chemotherapy (‘chemoresistant’) if a CR or PR has not been achieved and the response has been satisfactorily assessed. ‘Previous line of chemotherapy’ refers to the most recent course of systemic treatment given to the patient prior to PEP‐C. The presence or absence of extranodal disease refers to disease status when starting treatment with PEP‐C. CR, complete response; PR, partial response. [file JHA2-3-1316-s002.docx]

**Supplementary table II**

Response to PEP-C for all patients and subdivided by patient characteristics found to be statistically significant in a multivariate logistic regression model. A patient is considered to be ‘refractory’ to PEP-C or to his previous line of chemotherapy (‘chemoresistant’) if a CR or PR has not been achieved and the response has been satisfactorily assessed. ‘Previous line of chemotherapy’ refers to the most recent course of systemic treatment given to the patient prior to PEP-C. The presence or absence of extranodal disease refers to disease status when starting treatment with PEP-C. CR=complete response. PR=partial response.

| **Patient characteristic** | **CR or PR to PEP-C** | **Refractory to PEP-C** | **Response to PEP-C not assessed** | **Total** |
| --- | --- | --- | --- | --- |
| Lymphoma  Low grade  High grade | 19  20 | 9  31 | 3  10 | 31  61 |
| Response to previous line of chemotherapy  CR or PR (chemosensitive)  Refractory (chemoresistant)  Missing data | 25  9  5 | 19  17  4 | 6  7  0 | 50  33  9 |
| No extranodal disease  Extranodal disease | 16  23 | 6  34 | 2  11 | 24  68 |
| Total | 39 | 40 | 13 | 92 |
